# Commentary: reimplantation versus root remodelling for connective tissue disorder patients

**DOI:** 10.1093/ejcts/ezac387

**Published:** 2022-07-27

**Authors:** Lars G Svensson

**Affiliations:** Department of Thoracic and Cardiovascular Surgery, Heart, Vascular, and Thoracic Institute, Cleveland Clinic, Cleveland, OH, USA

**Keywords:** Connective tissue disorder, Remodelling, Reimplantation, Aortic valve repair

The AVIATOR Registry article written by Chauvette *et al.* [1] is an interesting article because it compares the outcomes of 2 different operations for preserving the aortic valve leaflets during aortic root surgery in patients with inherited connective tissue disorder (CTD). Ideally, patients would be randomized to either 2 operations, but it is unlikely that enough surgeons would have equipoise to do that in patients with CTD. Thus, this article is probably the best comparison group of patients (*N* = 237), particularly since variations of annular support were tried for the remodelling operation.

The principle of aortic valve repair, depending on if the valve is trileaflet or bileaflet (bicuspid), is different. For a trileaflet valve, the principle is to restore apposition of the leaflets. For bicuspid valves, it is to stretch the leaflets along the line of apposition. Thus, for a trileaflet reimplantation operation, it is to bring the leaflets together again. For a bicuspid valve, it is to keep tension on the leaflets. Hence, for reimplantation, I reduce the annulus using a Hegar dilator for the normalized size for body surface area, whereas for a bicuspid valve reimplantation or remodelling, I measure the maximal length of the leaflets to choose the graft diameter, usually the largest available, and brace the annulus [2–7]. As evidence of our approach to reimplantation of the valve, we recently presented our series of 214 patients with CTD and reimplantation at the Western Thoracic Surgical Association annual meeting in June 2022; our results show no operative deaths and 97% freedom from reoperation at 10 years. Having reoperated on a number of remodelling operations in CTD patients with splaying of the annulus, we no longer use remodelling for CTD patients but do use it for bicuspid valves. These similar outcomes were reported by Chauvette and colleagues in this article and also reported by us, with a high reoperation rate for CTD [4]. Whether the long-term results will be equivalent with annular support remains to be seen. I encourage the authors to follow their patients long-term to update us on their excellent series.
